# Coronin 6 promotes hepatocellular carcinoma progression by enhancing canonical Wnt/beta-catenin signaling pathway

**DOI:** 10.7150/jca.62873

**Published:** 2021-11-04

**Authors:** Jing Zhang, Pengfei Li, Ting Li, Zhiqin Zhou, Huiling Wu, Lei Zhang

**Affiliations:** 1 Department of Plastic and Aesthetic Center, The First Affiliated Hospital, Zhejiang University School of Medicine, Hangzhou, 310003, China.; 2 School of Medicine, Zhejiang University, Hangzhou, 310058, China.

**Keywords:** hepatocellular carcinoma, Coronin 6, Wnt/beta-catenin signaling pathway

## Abstract

Hepatocellular carcinoma (HCC), which is one of the most commonly diagnosed cancers, accounts for a large majority of cancer-related mortality worldwide. Although various genes have already been found to play vital regulatory roles in HCC progression, the pathological mechanism is still not well-understood. In this study, we find Coronin 6 (CORO6) is highly expressed in HCC samples with higher grades and is correlated with poor patient outcomes. CORO6 depletion significantly impairs the cell survival, migratory and invasive abilities of HCC cells. Pathway analysis and reporter assay reveal that Wnt signaling is enhanced by CORO6 in HCC cells. Moreover, *WNT10B* is identified as a target gene of CORO6. *In vivo* experiments suggest that knockdown of CORO6 inhibited the tumor growth. Importantly, expression of the key WNT target genes that are involved in cell cycle regulation and tumorigenesis, is downregulated in the absence of CORO6. Collectively, our results uncover a novel function of CORO6 in HCC progression and prove that the activation of WNT signaling is responsible for the tumor-promoting role of CORO6, which may offer a new target for therapeutic gain of treating HCC.

## Introduction

Worldwide, hepatocellular carcinoma (HCC) is amongst the most common causes of death resulted from cancers 1. A large proportion of HCC has been reported to be originally derived from liver diseases which are chronic with long-lasting liver inflammation, fibrosis as well as dysregulated hepatocyte regeneration. These aberrations induce a series of events at the genetic and epigenetic levels, which ultimately leads to the hyperactivation of cell survival and aggressiveness, like migration and invasion, of HCC cells 2. Moreover, infection by HBV accounts for the most cause of hepatocellular carcinoma 3. Although imaging techniques greatly contribute to the diagnosis of HCC and systematic therapies aid to the treatment of HCC patients, pathological markers as well as druggable targets are required for the improvement of diagnosis and therapeutic purposes 4.

As a pivotal pathway to trigger HCC progression, mutations of Wnt signaling components are proved to be not uncommon in HCC patients 5. Around 11-41% of HCC patients harbor *CTNNB1*-activating mutations, while about 5-19% of HCC specimens harbor *AXIN1* mutations 6, 7. The *CTNNB1* and *AXIN1* gene encode β-catenin and AXIN1 protein, respectively, both of which are essential components of the canonical Wnt signaling. Wnt pathway is activated when Wnt ligands bind to the cell surface receptors consisting of frizzled and co-receptors including LDL Receptor Related Protein 5 or 6 (LPR5/6). When Wnt pathway is in an inactive status, β-catenin is phosphorylated and ubiquitinated by a complex for destruction, which mainly contains AXIN, APC, GSK-3 and the β-TrCP which is an E3 ligase. Upon the interaction between Wnt ligands and receptors, the complex responsible for destruction is silenced, leading to the upregulation of non-phosphorylated β-catenin in the cytosolic part of cells and the nuclear translocation of β-catenin. Afterwards, β-catenin interacts with LEF/TCF protein to promotes the expression of downstream genes including *CCND1*, *CD44* as well as *c-Myc* 8. Given the findings that Wnt pathway is involved in the maintenance of normal functions of stem cells such as intestine stem cells, systematic targeting Wnt signaling for the treatment of HCC may cause so-called on-target side effects 9. Thus, identifying and targeting tissue specific expressed regulators of Wnt signaling can retain the inhibitory effects on Wnt pathway and, at the same time, bypass the side effects resulted from targeting Wnt pathway systematically.

Coronin 6 (CORO6) is one of the seven coronin proteins that have been identified in mammals. Coronins were reported to regulate actin dynamics through the binding to F-actin 10. CORO6 is mainly expressed in adult muscles and is pivotal for the modulation of receptor-associated cytoskeleton by anchoring acetylcholine receptors onto the actin cytoskeleton 11. Nevertheless, the role of CORO6 in cancer including HCC progression is ill-studied. Here, we validate that mRNA and protein expression of *CORO6* is upregulated in HCC specimens classified into more aggressive grades and CORO6 is an indicator for unfavorable prognosis. Loss-of-function experiments show that CORO6 depletion suppresses cell viability, migration, and invasion. Interestingly, Wnt pathway is enriched as the target of CORO6. Mechanistically, *WNT10B* is positively regulated by CORO6. Furthermore, we prove that CORO6 knockdown attenuates HCC tumor growth in mice. Importantly, expression of Wnt target genes is greatly downregulated in tumors with CORO6 depletion, indicating Wnt signaling is mitigated upon the knockdown of CORO6 in HCC. Together, our results uncover that CORO6 may serve to be a candidate biomarker and promising therapeutic target for HCC patients.

## Methods

### Bioinformatic data analyses

RNA-seq data (normal: n=50, tumor: n=369) were obtained from TCGA-LIHC database via link https://portal.gdc.cancer.gov/. Raw count data normalization and differential analyses were carried out by using R edgeR (version 3.30.3). The threshold was set as |log2FC (fold change) |>1 and FDR<0.05. Corresponding clinical data were obtained from UCSC Xena (http://tcga.xenahubs.net/) and analyzed for survival probability by applying the R survival package (version 3.1-12).

### Cells

HCC cell lines Hep3B, Huh7, SMMC-7721, HepG2 and PLC/PRF/5, as well as normal human liver cell line L-02 were purchased form Xiamen Immocell Biotechnology Co.,Ltd. and were maintained in high glucose Dulbecco's Modified Eagle medium (DMEM; Thermo Fisher Scientific; Catalog number: 11965092) supplemented with 10% heat inactivated fetal bovine serum (FBS; Gibco; Catalog number: 12483020), 2mM L-glutamine and 100 U/ml penicillin/streptomycin (Gibco; Catalog number: 15140122). All these cells were kept at 37°C in a humidified-air 5% CO_2_ atmosphere.

### Construction of plasmid

Standard cloning protocols were applied in this study using the ClonExpress Ultra One Step Cloning Kit (Vazyme; Catalog number: C115-01). The coding region of CORO6 was amplified with the aid of the primers listed in Table 1, after which, the purified PCR product was digested and ligated to PCDH-EF1a-mcs-T2A-puro backbone, leading to the generation of CORO6 ectopic expression construct.

Plasmids expressing CORO6-specific shRNAs were assembled by inserting the annealed oligonucleotides (Table 1) into digested acceptor vector pLKO.1-TRC.

### Quantitative real-time PCR (RT-qPCR)

Quantitative real-time PCR was performed for determining the mRNA levels of indicated genes. In brief, RNA was extracted by using Trizol reagent (Thermo Fisher Scientific; Catalog number: 15596026) according to the manufacturer's recommendations. Afterwards, 100 ng - 1 μg of total RNA was applied for cDNA synthesis with ReverTra Ace qPCR RT Kit (TOYOBO; Catalog number: TYB-FSQ-101) following the manufacturer's instructions. Next, the various target genes were amplified with Real-time PCR by using SYBR Select Master Mix (Thermo Fisher; Catalog number: 4472919) and the CFX Connect Detection System (Bio-Rad). The primer pairs were specified in Table 2. The relative quantification of RNA expression was calculated via the 2^-ΔΔCt^ method and normalized to 18s rRNA.

### MTT assay

3-(4,5-Dimethylthiazol-2-yl)-2,5-diphenyltetrazolium bromide (MTT; Yeasen; Catalog number: 40206ES76) assays were carried out for the detection of cell viability. Hep3B and SMMC-7721 cells were seeded into the wells of 96-well plates at a density of 1 × 10^3^ cells per well. 20 μl of 5 mg/ml MTT dissolved in PBS was directly added into each well at the indicated time points and subsequently the supernatant was substituted with 100 μl dimethyl sulfoxide (DMSO) after 4 h incubation at 37°C. Absorbance values at 490 nm were measured on a SpectraMax Absorbance Reader (Molecular Devices, San Francisco, CA, USA).

### Colony formation assay

Hep3B and SMMC-7721 cells with CORO6 depletion were seeded into wells of 6-well plates at the density of 1.5 × 10^3^ per well. After approximately two weeks sub-culturing in regular culture medium, the colonies were fixed with 100% methanol and subsequently stained with crystal violet for 15 min at room temperature (RT).

### Flow cytometry

Cell cycle and apoptosis analyses were performed as follows. Briefly, cells were washed once with PBS, typsinized and resuspend. Next, the single cell suspension was stained with propidium iodide (PI; Vazyme, Nanjing, China; Catalog number: A211-02) or together with Annexin-V-fluorescein isothiocyanate (Annexin V-FITC; Vazyme; Catalog number: A211-02) following the manufacturer's indications. Flow cytometry analyses were done by using a NovoCyte flow cytometer (ACEA, San Diego, CA, USA; Catalog number: 1300). At least 10,000 viable single cells were acquired for each sample. Finally, the results were analyzed with software NovoExpress 1.4.1 (ACEA).

### Assays to determine cell migratory and invasive capacities

In terms of assays for testing cell migratory abilities, cells were transfected with indicated plasmids 24 h prior to trypsinization and seeding at a density of 3 × 10^4^ in the top chamber of 24-well transwell plates (Corning) that consist of membranes with 8 μm-pores. At approximately 24 h after seeding, cells that migrated to the lower flat were washed, fixed by PFA and stained with the methanol-dissolved crystal violet and photographed. Just before invasion assays, the same chambers as mentioned above were coated with matrigel (BD Biosciences, Catalog number: 354234). Subsequently, HCC cells, after 24 h post transfection, were seeded at a density of 3 × 10^4^ in the aforesaid matrigel-coated chamber. Upon 48 h incubation, cells that migrated to the lower flat were washed, fixed by PFA and stained with the methanol-dissolved crystal violet and photographed.

### Dual luciferase reporter assays

Hep3B or SMMC-7721 cells, at a confluence of 70%, were transfected by TOP-flash or FOP-flash reporter construct, Renilla luciferase expressing construct and CORO6 or control construct. Upon 48 h transfection, luciferase activities were measured from cell lysates by the ONE-Glo Luciferase Assay Kit (Proteintech; Catalog number: E6110). Relative luciferase activity was calibrated by luciferase activity from substrate for Renilla.

### Western blotting

Cells were lysed with RIPA buffer freshly added 1 × complete protease inhibitor (Roche; Catalog number: 11836153001). DC™ protein assay kit (Bio-Rad) was applied for the determination of protein concentrations. Equal amounts of proteins were loaded and separated by sodium dodecyl sulfate polyacrylamide gel electrophoresis (SDS-PAGE). The resolved proteins were subsequently transferred onto 45-μm polyvinylidene difluoride (PVDF) membrane (Merck; Catalog number: IPVH00010). Afterwards, a blocking step was performed by incubating the membranes with 5% non-fat dry milk at RT for 1 h. Next, the resulting membranes were probed with antibodies directly raised against CORO6 (Proteintech; Catalog number: 17243-1-AP), CCND1 (Proteintech; Catalog number: 26939-1-AP), c-Myc (Proteintech; Catalog number: 10828-1-AP), AXIN2 (Abcam; Catalog number: ab109307), and GAPDH (Proteintech; Catalog number: 10494-1-AP) diluted 1:1000 in TBST supplemented with 5% BSA, respectively. After an overnight incubation at 4°C and washing steps, blots were incubated with secondary antibodies against mouse IgG (Proteintech; Catalog number: SA00001-1) and rabbit IgG (Proteintech; Catalog number: SA00001-2), and the signals were detected.

### Immunohistochemical (IHC) staining and evaluation

The tissue microarray slide consisting of adjacent normal tissues (ANT) and matched HCC tumor tissues (Shanghai Outdo Biotech Co., Ltd; Catalog number: HLivH150CS05) were baked at 65°C for 2 h. Then paraffine was removed by placing slides in xylene for three times, followed by placing in 100% ethanol twice. Peroxidase activity was inhibited by hydrogen peroxide. Then the slide was rehydrated in 96%, 70% and 50% Ethanol, respectively. Afterwards, antigen retrieval was carried out after washing slides with PBST for 5 min. The slide was then slowly cooled down to RT, followed by 3 times washing with PBST. Primary antibody against CORO6 (Proteintech; Catalog number: 17243-1-AP) diluted (1:100) in 1% BSA (dissolved in PBST) was used to incubate the slide overnight. After the washing steps, the slide was incubating with 1:200 diluted biotinylated secondary antibody (DAKO) for 30 min at RT. After 3 times washing with PBST, slides were subjected to the incubation with Vectastain complex (Vector Laboratories) for 30 min. Subsequently, the slide was washed with PBST thrice and developed by DAB. Next, slides were counterstained with Mayers Haematoxylin (Sigma-Aldrich) for 45 s and dehydrated. Finally, Entellan was applied to mount the slides. Images were captured using a panoramic scanner PANNORAMIC (3DHISTECH Ltd., Budapest, Hungary) and software CaseViewer2.4 (3DHISTECH Ltd.). In order to quantify the results of IHC, Software AIpathwell (Servicebio, Wuhan, China) was used to analyze the staining intensity and rate of positive cells. Each specimen was assigned a score according to the intensity of cytoplasmic staining (no staining = 0; weak staining = 1, moderate staining = 2 and strong staining = 3). The CORO6 protein level was quantified using the formula: level = intensity score ×positive rate × 100.

### Animal studies

All the mice assays were performed following a protocol of Animal Care and Use Committee of Zhejiang University. For *in vivo* cell survival assays, 5 × 10^6^ Hep3B cells with stable CORO6 knockdown or empty vector were injected subcutaneously in the 6-week-old BalB/C nude mice for 32 days (n=6). The volumes of tumors were monitored every 4 days with calipers from 16 days post-injection. All mice were euthanized at 32 days after injection, after which tumors were collected and photographed. Tumor weight was determined at 32 days post-injection.

### Statistical analyses

The analyses were done with the help of GraphPad Prism on the data derived from independent biological replicates. All quantitative data were exhibited as mean ± s.d. P < 0.05 was determined to be statistically significant.

## Results

### *CORO6* mRNA is increased during HCC progression and is correlated with poor prognosis

**Figure 1:**
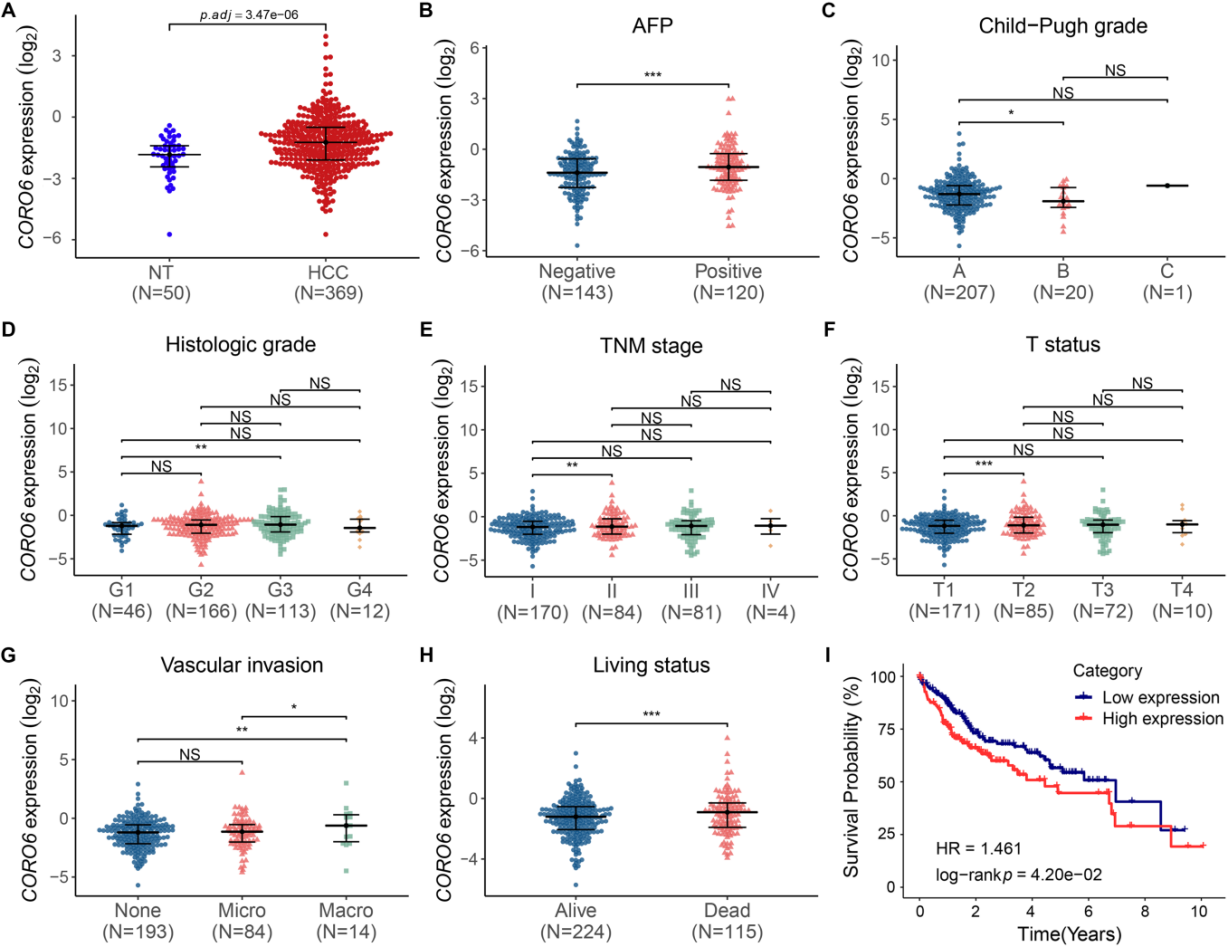
** CORO6 mRNA is increased during HCC progression and is correlated with poor prognosis.** (A) Comparison of *CORO6* mRNA level in samples from normal tissues (n=50) and primary HCC tumor tissues (n=369). The RNA-seq raw data were derived from the TCGA-LIHC database (https://portal.gdc.cancer.gov/). (B - H) Comparisons of *CORO6* mRNA expression in HCC tumor tissues classified by AFP level (B), Child-Pugh grade (C), histologic grade (D), TNM stage (E), T status (F), vascular invasion (G) and living status (H). Significance was calculated between indicated groups by using Mann-Whitney test. (I) Survival probability of patients stratified by low and high *CORO6* mRNA levels. *P < 0.05; **P <0.01; ***P < 0.001; NS: not significant.

To study the function of CORO6 in HCC, we started with testing the level of *CORO6* mRNA by applying publicly available databases. By analyzing the transcriptome data from normal liver and HCC tissues, we found *CORO6* was significantly increased in samples from HCC patients, when compared with normal liver tissues (Figure 1A). In addition, *CORO6* was more expressed in AFP positive patients than AFP negative HCC patients whose prognosis is generally better (Figure 1B) 12. Furthermore, *CORO6* was determined to be upregulated in Child-Pugh grade B subgroup, in comparison of grade A subgroup in which the HCC is less aggressive (Figure 1C) 13. Importantly, when grade G3 compared with grade G1 in terms of histologic stage, stage II compared with stage I in terms of TNM stage, and status T2 compared with status T1 in terms of T status, expression of *CORO6* was significantly increased (Figure 1D - 1F). The correlation between clinicopathological variables and *CORO6* expression in HCC was summarized in Table 3. Since vascular invasion is a critical parameter for evaluating the aggressiveness of HCC 14, CORO6 expressed was then analyzed to check its correlation with vascular invasion. Interestingly, *CORO6* was significantly more expressed in HCC patients who are diagnosed to form macro vascular invasion, in comparison to those with micro vascular invasion as well as to patients without any detected invasion (Figure 1G). Moreover, association between *CORO6* expression and patient prognosis was assessed. In line with the previous data, the *CORO6* mRNA level in samples from HCC patients who were not alive after 5 years was higher than in those who were alive (Figure 1H). Furthermore, lower survival probabilities, as shown by a Kaplan-Meier plot, were observed in patients with higher *CORO6* levels (Figure 1I). Collectively, *CORO6* is found to be higher expressed in HCC samples with grades that are more aggressive and is correlated with unfavorable outcomes of HCC patients.

### CORO6 protein is upregulated in HCC specimens and cell lines

**Figure 2:**
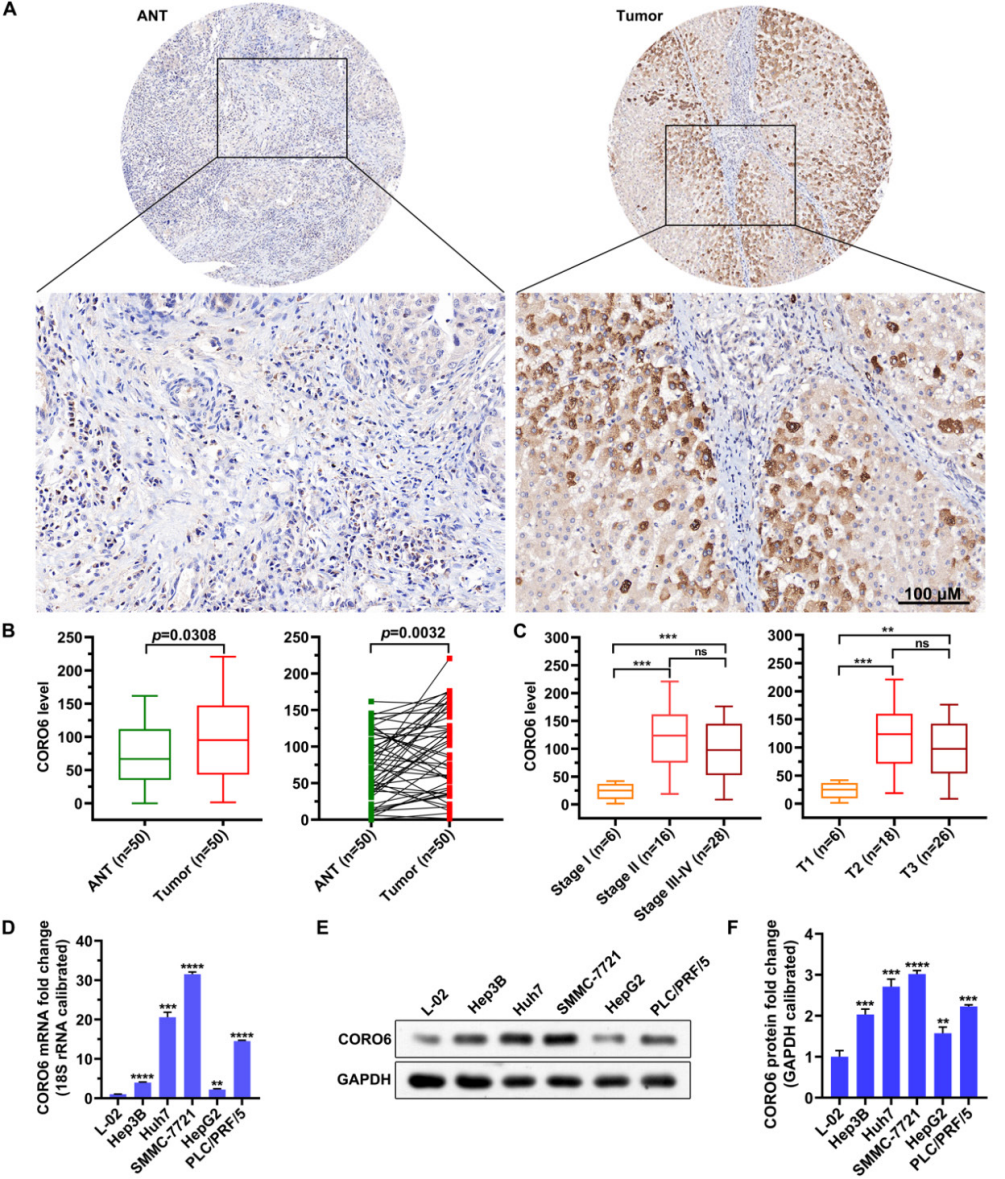
** CORO6 protein is upregulated in HCC specimens and cell lines.** (A) Immunohistochemical (IHC) staining of CORO6 protein in adjacent normal tissues and tumor tissues on the tissue microarray. Representative images are shown. (B) Statistical comparison of CORO6 protein level in IHC-stained adjacent normal tissues (n=50) and tumor tissues (n=50). Significance analyses were performed between indicated groups by using unpaired Mann-Whitney test (left panel) and paired Wilcoxon matched-paired signed rank test (right panel), respectively. (C) Statistical analyses of CORO6 protein level in IHC-stained tumor tissues (n=50) classified by TNM stage (left panel) and T status (right panel). Significance was calculated between indicated groups with Mann-Whitney. (D) Quantification of *CORO6* mRNA level in normal liver cell (control) as well as HCC cells via RT-qPCR. Significance analyses were performed between indicated groups by using multiple comparisons followed by Turkey test. (E and F) Western blotting analysis of CORO6 protein level in normal liver cell and HCC cells (E). Significance was calculated between indicated groups with multiple comparisons followed by Turkey test (F). **P <0.01; *** < 0.001; ****P < 0.0001; ns: not significant.

To further investigated if CORO6 protein was dysregulated in HCC, we proceeded by performing immunohistochemistry (IHC) staining in adjacent normal tissues (ANT) and HCC tumor tissues. We detected that CORO6 protein was significantly higher expressed in tumor tissues compared with adjacent normal tissues (Figure 2A and 2B). Moreover, paired comparison indicated that CORO6 protein was more expressed in tumor tissue than that in adjacent normal tissues most of the paired HCC patients (Figure 2B). Strikingly, amongst tumor tissues, protein level of CORO6 was dramatically upregulated in Stage 2 and Stage 3-4 in comparison with Stage 1 (Figure 2C). Similarly, in another classification method, CORO6 protein was more expressed in T2 and T3 stages compared with T1 stage (Figure 2C). Next, we moved to examine the level of CORO6 in normal liver cells as well as HCC cell lines. In line with data from patients with HCC, *CORO6* mRNA and protein expression was significantly more expressed in HCC cell lines (Hep3B, Huh7, SMMC-7721, HepG2 and PLC/PRF/5) compared with normal liver cells L-02 (Figure 2D-2F). Similarly, Table 4 shows that overexpression of CORO6 was significantly associated with pathologic T (*P* = 0.0183, Table 4). Taken together, CORO6 protein is positively associated with the unfavorable outcomes of HCC patients and is decreased in aggressive HCC cell lines.

### CORO6 depletion inhibits HCC cell survival, migratory and invasive abilities

**Figure 3:**
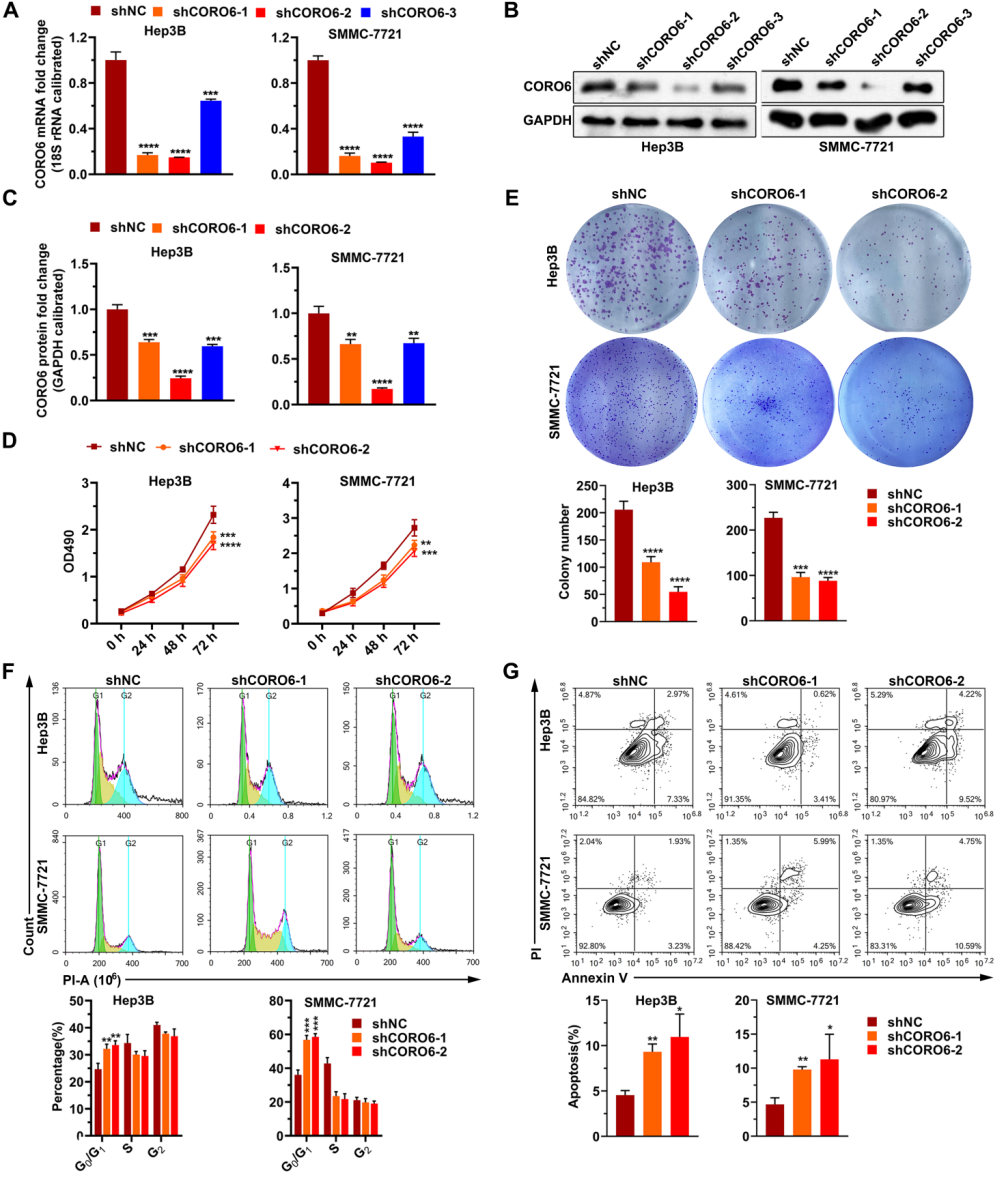
** CORO6 depletion suppresses HCC cell proliferation, and promotes apoptosis.** (A - C) Knockdown efficiency validation of three independent shRNAs targeting *CORO6* by RT-qPCR (A) and western blotting (B) in Hep3B and SMMC-7721 cells. Significance analyses of CORO6 protein level were performed between negative control groups (shNC) and groups treated with different shRNAs targeting *CORO6* (shCORO6-1, shCORO6-2 and shCORO6-3) by using unpaired Student's t-tests (C). (D) Assessment of cell viability by MTT assay upon *CORO6* knockdown in Hep3B and SMMC-7721 cells. Significance was calculated between negative control groups (shNC) and groups treated with different shRNAs targeting *CORO6* (shCORO6-1 and shCORO6-2) by using unpaired Student's t-tests. (E) Colony formation assay of Hep3B and SMMC-7721 cells upon CORO6 depletion. Representative images of colonies are exhibited in the upper panel. Significance was calculated between negative control groups (shNC) and groups treated with different shRNAs targeting *CORO6* (shCORO6-1 and shCORO6-2) by using unpaired Student's t-tests (lower panel). (F) Cell cycle analyses of Hep3B and SMMC-7721 cells with CORO6 knockdown. Representative images of FACS analyses are exhibited in the upper panel. Significance analyses were carried out between negative control groups (shNC) and groups treated with different shRNAs targeting *CORO6* (shCORO6-1 and shCORO6-2) by using unpaired Student's t-tests (lower panel). (G) Detection of cell apoptosis with Annexin V/PI staining assays in CORO6 knockdown Hep3B and SMMC-7721 cells. Representative images of FACS analyses are shown in the upper panel. Significance analyses were carried out between negative control groups (shNC) and groups treated with different shRNAs targeting *CORO6* (shCORO6-1 and shCORO6-2) by using unpaired Student's t-tests (lower panel). *P < 0.05; **P <0.01; ***P < 0.001; ****P < 0.0001.

**Figure 4:**
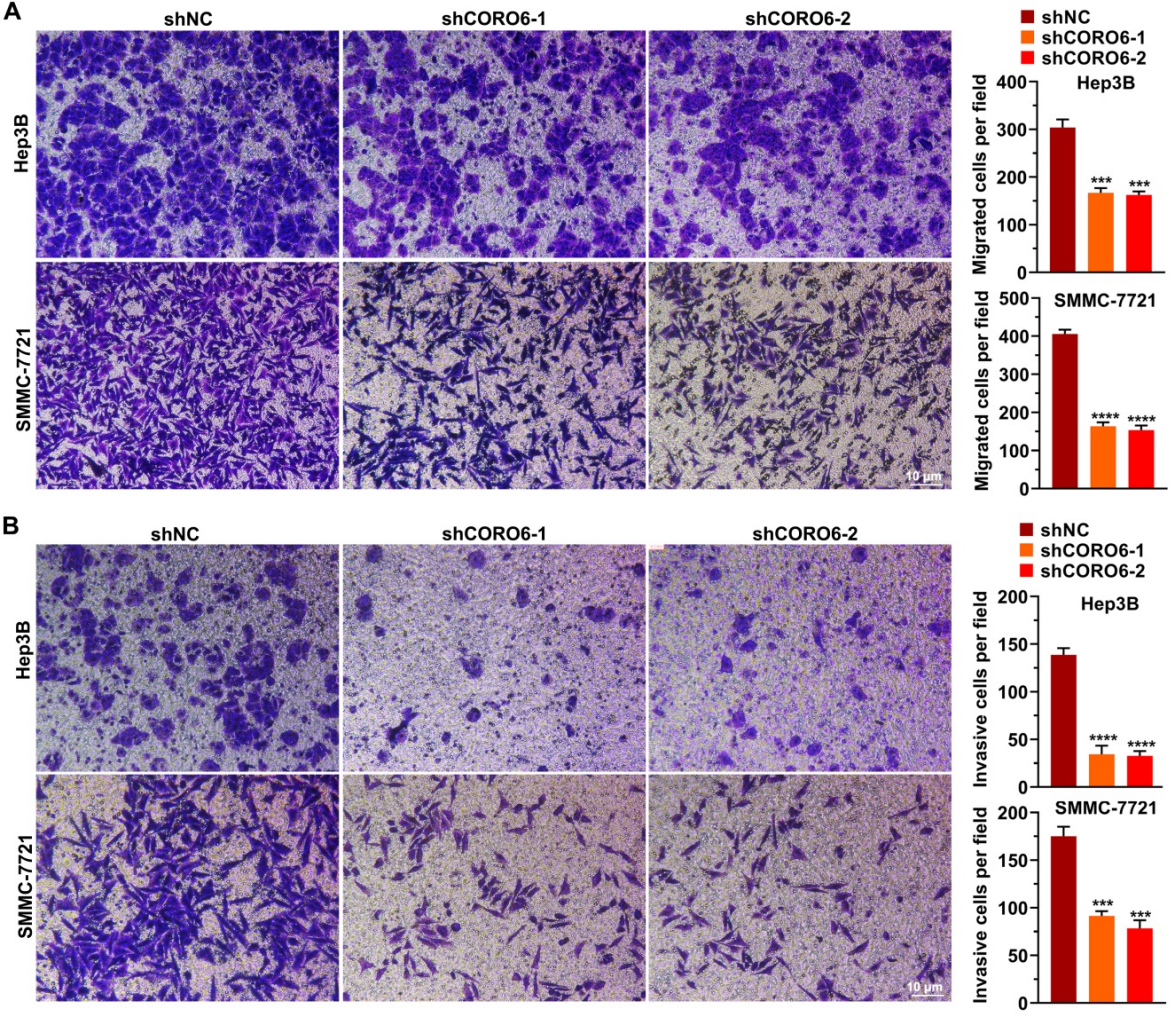
** CORO6 depletion inhibits migratory and invasive abilities.** (A) Assessment of cell migratory ability in Hep3B and SMMC-7721 cells with CORO6 depletion. Representative images are shown in the left panel. Significance was calculated between negative control groups (shNC) and groups treated with different shRNAs targeting *CORO6* (shCORO6-1 and shCORO6-2) by using unpaired Student's t-tests (right panel). (B) Evaluation of cell invasive ability in Hep3B as well as SMMC-7721 cells upon CORO6 knockdown. Representative images are shown in the left panel. Significance was calculated between negative control groups (shNC) and groups treated with different shRNAs targeting *CORO6* (shCORO6-1 and shCORO6-2) by using unpaired Student's t-tests (right panel). ***P < 0.001; ****P < 0.0001.

To further mine the function of CORO6 in HCC cell malignancy, we first depleted CORO6 in two HCC cell lines Hep3B and SMMC-7721 by three shRNAs targeting various sequences. As seen in Figure 3A-3C, for these two HCC cell lines, mRNA and protein expression of *CORO6* was successfully decreased upon the transduction of three shRNAs targeting CORO6. ShRNA No. 1 and No.2 were applied for further experiments due to their most potent knockdown efficiency. MTT assay results showed that depletion of CORO6 dramatically impaired the cell viability of HCC cells (Figure 3D). Additional colony formation experiments also indicated that loss of CORO6 resulted in less colonies formed by HCC cells (Figure 3E). Since cell viability is closely correlated with cell cycle progression and apoptosis, we checked whether cell cycle was affected by CORO6 knockdown. As expected, upon CORO6 depletion, the proportion of G0/G1 phase of HCC cells was increased (Figure 3F). In agreement with this result, the proportion of apoptotic cells were enhanced in CORO6-depleting HCC cells (Figure 3G). Next, cell migration as well as invasion was evaluated by transwell experiments. Importantly, we validated that CORO6 knockdown resulted in the decrease of cell migratory and invasive capabilities (Figure 4A and 4B). Collectively, CORO6 is proved to contribute to the survival of HCC cells, which is achieved by impairing the progression of cell cycle and enhancing cell apoptosis. Furthermore, the cell migration and invasion are inhibited when CORO6 is absent.

### CORO6 activates Wnt signaling by promoting *WNT10B* expression

**Figure 5:**
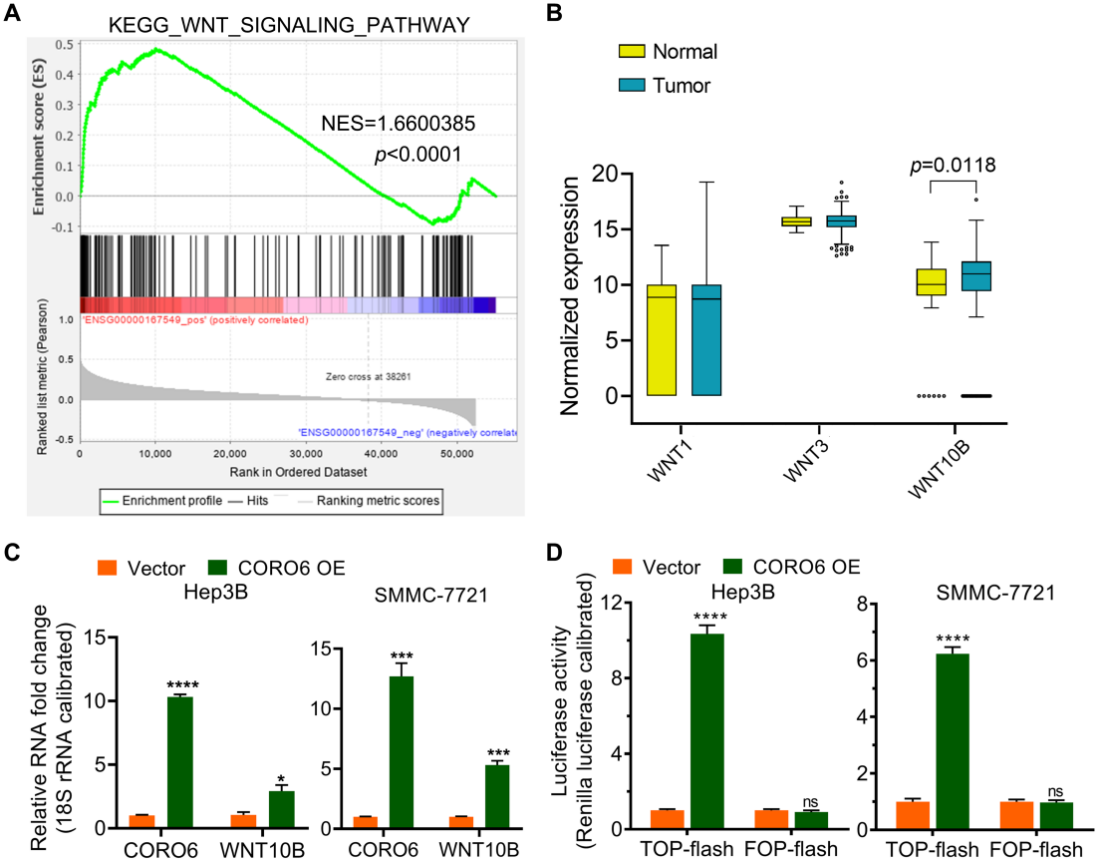
** CORO6 activates Wnt signaling by promoting WNT10B expression.** (A) Gene set enrichment analysis (GSEA) for RNA-seq data from TCGA-LIHC database. (B) Statistical comparison of *WNT1*, *WNT3* and *WNT10B* mRNA expression between normal and tumor samples from TCGA-LIHC database by using unpaired Student's t-tests. (C) Detection of *WNT10B* level via RT-qPCR in Hep3B and SMMC-7721 cells with CORO6 ectopic expression by using unpaired Student's t-tests. (D) TOP-flash/FOP-flash dual luciferase assay for detecting Wnt/b-catenin-driven downstream transcriptional activity. FOP-flash activity was set as a negative control. Unpaired Student's t-tests were performed to test the statistical significance. *P < 0.05; ***P < 0.001; ****P < 0.0001; ns: not significant.

To further uncover the molecular mechanism by which CORO6 promotes HCC progression, KEGG pathway enrichment analysis was carried out. Significantly, Wnt signaling pathway was enriched as the top pathway that correlated with CORO6 (Figure 5A). We hypothesized that CORO6 may regulate the expression of Wnt ligands, thereby enhancing the transduction of Wnt signaling. To this end, we checked if there was differential expression of Wnt ligands including *WNT1*, *WNT3* and *WNT10B* among normal liver and HCC tumor tissues. To our surprise, only *WNT10B* was shown to be significantly upregulated in tumor specimens in comparison of liver specimens derived from normal donors, which indicated that possible correlation between CORO6 and WNT10B in the regulation of HCC progression (Figure 5B). As expected, CORO6 overexpression upregulated the mRNA level of *WNT10B* (Figure 5C). Moreover, the downstream reporter of Wnt pathway was applied to measure the activity of Wnt pathway. As shown in Figure 5D, TOP-flash reporter activity, indicating the enhancement of Wnt signaling, was strikingly enhanced in HCC cell ectopically expressing CORO6, while the negative control FOP-flash luciferase activity was unaffected. Together, our results suggested that CORO6 may augment Wnt signaling by upregulating the level of *WNT10B*.

### CORO6 knockdown inhibits HCC cell growth *in vivo* by attenuating Wnt pathway

**Figure 6:**
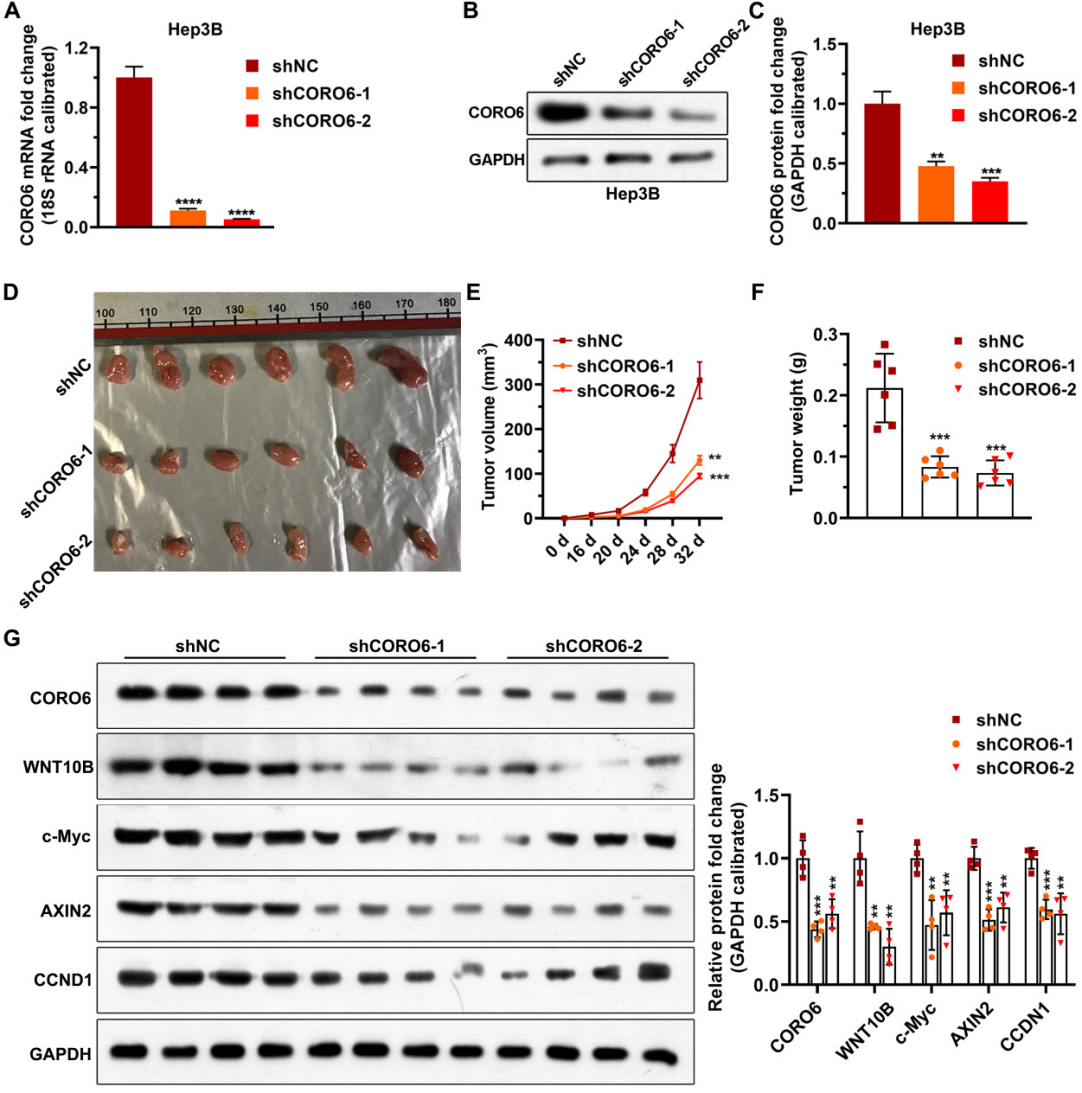
** CORO6 knockdown inhibits HCC cell growth *in vivo* by attenuating Wnt pathway.** (A) Validation of CORO6 knockdown efficiency by qPCR in Hep3B stable cell lines. Analysis was carried out using unpaired Student's t-tests. (B and C) Confirmation of CORO6 knockdown efficiency by western blotting in Hep3B stable cell lines (B). Analysis was performed by unpaired Student's t-tests (C). (D) Representative images of tumors isolated from mice that are subjected to Hep3B cells with CORO6 depletion. At the top of the image is a scale, the unit is cm. (E and F) Quantification regarding volume of tumors (E) as well as weight (F) in mice (n = 6) that were injected with CORO6 depleted Hep3B cells. Unpaired Student's t-tests were done between the control group (shNC) and CORO6 knockdown groups (shCORO6-1 and shCORO6-2), respectively. (G and H) Western blotting assessment of protein levels of Wnt target genes in tumors formed by Hep3B stable cells with CORO6 knockdown (G). Unpaired Student's t-tests were carried out between the control group (shNC) and CORO6 knockdown groups (shCORO6-1 and shCORO6-2), respectively (H). **P <0.01; ***P < 0.001; ****P < 0.0001.

To investigate the biological role of CORO6 on cell survival *in vivo*, we subcutaneously injected Hep3B stable cells, which were generated by lentiviral infection, in mice. In agreement with what we got from *in vitro* experiments, depletion of CORO6 significantly decreased the growing ability of Hep3B cells in mice (Figure 6A-6F). To confirm that Wnt signaling is perturbated by the loos of CORO6 in tumors formed by HCC cells, we detected the levels of Wnt downstream genes that reported to play roles in cell cycle regulation and tumorigenesis 15-17. Interestingly, we observed that protein levels of WNT10B as well as several Wnt target genes including c-Myc, AXIN2, CCND1 were obviously downregulated in tumors generated from Hep3B cell with CORO6 depletion (Figure 6G and 6H). Taken together, CORO6 knockdown leads to the inhibition of cell growth of HCC cells *in vivo* by mitigating Wnt pathway.

## Discussion

Coronin proteins have been proved to regulate actin dynamics in mammal cells 15-17. So far, however, the functions of CORO6 are generally poorly-understood. Here, by applying datamining strategies, we find that *CORO6* mRNA is significantly higher expressed in HCC patients with more malignant clinical traits and correlated with poor outcomes of HCC patients (Figure 1). Although we confirmed that CORO6 protein expression is upregulated in HCC tumors classified into more malignant stages, the *CORO6* mRNA differential analyses in our own patient cohort still need to be conducted in the future as well, to better consolidate the expression pattern of *CORO6* in HCC. This can also help with further testing if *CORO6* is eligible to serve as a biomarker for HCC patient diagnosis.

Loss-of-function experiments demonstrate that depletion of CORO6 decreases the viability, migratory and invasive capacities of HCC cells *in vitro* (Figure 3 and 4). Since CORO protein have been reported to interact with F-actin within cells 10, we could not exclude the possibility that CORO6 may enhance the rearrangement of cytoskeleton of cells, thereby promoting the cell migratory and invasive abilities. So further rescue experiments may be performed to check whether F-actin ectopic expression can restore the mitigation of cell migration and invasion resulted from CORO6 knockdown.

Through datamining and experimental analyses, we reveal that Wnt pathway is activated by CORO6 ectopic expression, which is achieved by upregulating the expression of *WNT10B* (Figure 5). Of note, epithelial-mesenchymal transition (EMT) is vital for epithelial cells to gain mesenchymal characteristics and highly invasive capacity 18. Considering the fact that Wnt signaling is one of the most pivotal signaling pathways that promote EMT 19, we aim to investigate whether the EMT process is affected by CORO6 in further studies.

Although *WNT10B* mRNA levels is found to be enhanced by CORO6 overexpression (Figure 5C), the mechanism by which CORO6 upregulates *WNT10B* expression is still undetermined. As CORO6 protein is mainly localized in the cytoplasm (Figure 2A), CORO6 may regulate the transcription of *WNT10B* indirectly. On one hand, CORO6 may modulate the levels of transcription factors, such as NFAT1/SMAD3, which are involved in the transcriptional regulation of *WNT10B* 20. Thus, analyses of proteome and interactome of CORO6 can be carried out to screen protein partners of interest that are involved in the transcriptional regulation of *WNT10B*. On the other hand, several microRNAs, including miR-148a and miR-156 21, 22, have been reported to decrease the mRNA level of *CORO6*. Therefore, the alteration of *WNT10B* mRNA level, resulted from CORO6 misexpression, may be due to the regulation of microRNAs targeting CORO6 mRNA. To further test this hypothesis, RNA sequencing of microRNAs upon CORO6 misexpression can be performed, thereby screening the candidate hits that are responsible for the regulation of *WNT10B*.

Collectively, we prove that CORO6 is a pro-oncogenic protein in HCC progression, which is accomplished by activating Wnt pathway. Targeting CORO6 may contribute to the clinical treatment of HCC.

## Figures and Tables

**Table 1:** Primers for construction of plasmid in this study.

Name	Sequence (5' to 3')	
shCORO6-1	Forward	ccgggcagggaaagtgcttagtattctcgagaatactaagcactttccctgcttttt
	Reverse	aattaaaaagcagggaaagtgcttagtattctcgagaatactaagcactttccctgc
shCORO6-2	Forward	ccgggacagcagcattcggtactttctcgagaaagtaccgaatgctgctgtcttttt
	Reverse	aattaaaaagacagcagcattcggtactttctcgagaaagtaccgaatgctgctgtc
shCORO6-3	Forward	ccggcggccctagaagcggacgaatctcgagattcgtccgcttctagggccgttttt
	Reverse	aattaaaaacggccctagaagcggacgaatctcgagattcgtccgcttctagggccg
CORO6 OE	Forward	ctagagctagcgaattcgccaccatgagcagacgtgtggttcg
	Reverse	tgccctcagcggccgcggatccgtccgtgccgtccaccagc

**Table 2:** RT-qPCR primers used in this study.

Gene	Sequence (5' to 3')	
*18S*	Forward	aggcgcgcaaattacccaatcc
	Reverse	gccctccaattgttcctcgttaag
*AXIN2*	Forward	cagcagtgtagatggaat
	Reverse	gggaaatgaggtagagac
*CCND1*	Forward	tcattgaacacttcctctc
	Reverse	tgaacttcacatctgtgg
*CORO6*	Forward	ctgtgctggatattgactg
	Reverse	aatctgccacaccatgat
*c-MYC*	Forward	gcgactctgaggaggaac
	Reverse	gtgatccagactctgacctt
*WNT1*	Forward	ccgatggtggggtattgt
	Reverse	agtaccagttgcagactctt
*WNT3*	Forward	gagaatacttcagcagaatggata
	Reverse	ctacggcgagaatcattacaa
*WNT10B*	Forward	atcctcaagcgcggtttc
	Reverse	tggctactgcgtgcatga

**Table 3:** Correlation between clinicopathological variables and CORO6 expression in HCC.

		CORO6 Expression		
	**Total (N=339)**	**High (N=142)**	**Low (N=197)**	**P-value ^a^**
**Age (year)**				0.933
< 65	208 (61.4%)	88 (62.0%)	120 (60.9%)	
≥ 65	131 (38.6%)	54 (38.0%)	77 (39.1%)	
**Gender**				0.214
Male	231 (68.1%)	91 (64.1%)	140 (71.1%)	
Female	108 (31.9%)	51 (35.9%)	57 (28.9%)	
**Family history of cancer**				0.746
NO	196 (57.8%)	84 (59.2%)	112 (56.9%)	
YES	98 (28.9%)	38 (26.8%)	60 (30.5%)	
Unknown	45 (13.3%)	20 (14.1%)	25 (12.7%)	
**TNM stage**				0.895
I	170 (50.1%)	68 (47.9%)	102 (51.8%)	
II	84 (24.8%)	36 (25.4%)	48 (24.4%)	
III	81 (23.9%)	36 (25.4%)	45 (22.8%)	
IV	4 (1.2%)	2 (1.4%)	2 (1.0%)	
**Histologic grade**				0.297
G1-G2	212 (62.5%)	85 (59.9%)	127 (64.5%)	
G3-G4	125 (36.9%)	57 (40.1%)	68 (34.5%)	
Unknown	2 (0.6%)	0 (0%)	2 (1.0%)	
**Ishak score**				0.276
0-4	124 (36.6%)	49 (34.5%)	75 (38.1%)	
5-6	74 (21.8%)	27 (19.0%)	47 (23.9%)	
Unknown	141 (41.6%)	66 (46.5%)	75 (38.1%)	
**Child-Pugh grade**				0.268
A	207 (61.1%)	82 (57.7%)	125 (63.5%)	
B-C	21 (6.2%)	7 (4.9%)	14 (7.1%)	
Unknown	111 (32.7%)	53 (37.3%)	58 (29.4%)	
**Vascular invasion**				0.536
None	193 (56.9%)	76 (53.5%)	117 (59.4%)	
Micro	84 (24.8%)	36 (25.4%)	48 (24.4%)	
Macro	14 (4.1%)	8 (5.6%)	6 (3.0%)	
Unknown	48 (14.2%)	22 (15.5%)	26 (13.2%)	
**Alpha fetoprotein**				0.146
Negative	143 (42.2%)	52 (36.6%)	91 (46.2%)	
Positive	120 (35.4%)	58 (40.8%)	62 (31.5%)	
Unknown	76 (22.4%)	32 (22.5%)	44 (22.3%)	
**Residual tumor**				0.165
R0	301 (88.8%)	125 (88.0%)	176 (89.3%)	
R1-R2	12 (3.5%)	8 (5.6%)	4 (2.0%)	
Unknown	26 (7.7%)	9 (6.3%)	17 (8.6%)	
**Living status**				0.0884
Alive	224 (66.1%)	86 (60.6%)	138 (70.1%)	
Dead	115 (33.9%)	56 (39.4%)	59 (29.9%)	
**Disease status**				0.816
NO	163 (48.1%)	69 (48.6%)	94 (47.7%)	
YES	132 (38.9%)	53 (37.3%)	79 (40.1%)	
Unknown	44 (13.0%)	20 (14.1%)	24 (12.2%)	

^a^ Chi-square test.

**Table 4:** Correlation between clinicopathological variables and CORO6 expression in HCC.

		CORO6 Expression		
	**Total (N=50)**	**High (N=25)**	**Low (N=25)**	**P-value ^a^**
**Age (year)**				0.602
< 65	46 (92.0%)	22 (88.0%)	24 (96.0%)	
≥ 65	4 (8.0%)	3 (12.0%)	1 (4.0%)	
**Gender**				1
Male	37 (74.0%)	18 (72.0%)	19 (76.0%)	
Female	13 (26.0%)	7 (28.0%)	6 (24.0%)	
**Pathological grade**				0.723
Ⅰ	5 (10.0%)	3 (12.0%)	2 (8.0%)	
Ⅱ	18 (36.0%)	9 (36.0%)	9 (36.0%)	
Ⅱ-Ⅲ	20 (40.0%)	11 (44.0%)	9 (36.0%)	
Ⅲ	6 (12.0%)	2 (8.0%)	4 (16.0%)	
Ⅲ-Ⅳ	1 (2.0%)	0 (0%)	1 (4.0%)	
**Pathologic T**				***0.0183***
T1	6 (12.0%)	0 (0%)	6 (24.0%)	
T2	18 (36.0%)	12 (48.0%)	6 (24.0%)	
T3	26 (52.0%)	13 (52.0%)	13 (52.0%)	
**Pathologic N**				0.602
N0	46 (92.0%)	22 (88.0%)	24 (96.0%)	
N1	4 (8.0%)	3 (12.0%)	1 (4.0%)	
**Pathologic M**				0.47
M0	48 (96.0%)	25 (100%)	23 (92.0%)	
M1	2 (4.0%)	0 (0%)	2 (8.0%)	
**Distant metastasis**				1
No	50 (100%)	25 (100%)	25 (100%)	

^ a^ Chi-square test. Statistically significant p values are given in bold italic.
